# Endogenous ligands of bovine FFAR2/GPR43 display distinct pharmacological properties

**DOI:** 10.3389/fcell.2025.1645031

**Published:** 2025-08-20

**Authors:** Tainara Cristina Michelotti, Valérie Lamothe, Frédéric Jean-Alphonse, Eric Reiter, Muriel Bonnet, Guillaume Durand

**Affiliations:** ^1^ INRAE, Université Clermont Auvergne, VetAgro Sup, UMR Herbivores, Saint-Genès-Champanelle, France; ^2^ Feed and Food Department, Bordeaux Sciences Agro, Gradignan, France; ^3^ INRAE, CNRS, Université de Tours, PRC, Nouzilly, France; ^4^ Inria, Inria Saclay-Ile-de-France, Palaiseau, France

**Keywords:** cell signaling, cell surface receptor, fatty acid, bioluminescence resonance energy transfer, lipid signaling, G protein-coupled receptor, bovine free fatty acid receptor 2

## Abstract

**Introduction:**

Free fatty acids (FFAs) have been identified as ligands for members of the G protein-coupled receptor (GPCR) family, called free fatty acid receptors (FFARs). Among these receptors, there is a particular interest in the physiological roles of FFAR2 and its potential use as a therapeutic target for various health disorders. Despite great progress in other species, pharmacological properties of the bovine FFAR2 (bFFAR2) are not fully understood. The aim of the current study was to evaluate how a selection of FFAs (C2:0 to C8:0, and branched FFAs) activate and regulate bFFAR2 signaling.

**Methods:**

We used HEK293A cells and BRET assays to measure Gαi/Gαq coupling and signaling, β-arrestin 2 recruitment, and receptor internalization/trafficking. SRE and NFAT-RE dependent transcription was assessed by luciferase reporter assay.

**Results and discussion:**

Results show that bFFAR2 presents a dual coupling to Gαi and Gαq and recruits β-arrestin 2 when stimulated with short and medium-chain FFAs up to eight carbons. Straight-chain FFAs with 4 to 7 carbons plus 3-methyl-butanoic acid showed the greatest potency to activate bFFAR2 upstream and downstream signaling, while C2:0, C3:0 and 2-methylpropanoic acid (2MP) were the least potent. 2MP exhibited minimal pharmacological activity towards β-arrestin 2, and although it induced receptor internalization, bFFAR2 trafficking to the early endosome was not observed. Overall, the number of carbons of straight-chain FFAs and methyl position of branched FFAs differentially regulates the activation of bFFAR2.

## 1 Introduction

Short- and medium-chain fatty acids (SCFAs and MCFAs) are carboxylic acids with aliphatic tails of 1–6 and 7–12 carbons, respectively (Schönfeld and Wojtczak, 2016). In ruminants, SCFAs account for most of the animal’s metabolizable energy (Bergman, 1990). Largely produced through fermentation in the rumen, SCFAs’ composition reflects the biodiversity of the rumen microbiota and the dietary composition (Hocquette and Bauchart, 1999; Resende Júnior et al., 2006). In addition to the major SCFAs, e.g., acetate, propionate, and butyrate, branched-chain SCFAs (BSCFAs) are notable for positively affecting both the rumen microbiota and dairy cow metabolism (Peirce-Sandner et al., 1985; Gouveia et al., 2024).

Together with the long-chain fatty acids (LCFAs, more than 12 carbons), SCFAs and MCFAs are energy-rich molecules that function not only as metabolic fuel but also exhibit various regulatory and signaling functions in metabolism (Calder, 2015; Schönfeld and Wojtczak, 2016). As signaling molecules, SCFAs are known, for example, to have anti-inflammatory effects through the inhibition of histone deacetylases (HDACs), while MCFAs have been established as selective PPARγ activators (Liberato et al., 2012; Li et al., 2018). In addition, free fatty acids (FFAs), which are non-esterified or unbound fatty acids, have been associated with the activation of specific members of the G protein-coupled receptor (GPCR) family, also known as free fatty acid receptors (FFARs) (Briscoe et al., 2003; Brown et al., 2003; Hirasawa et al., 2005).

Among the different FFARs that have been identified, there is particular interest in the physiological roles of FFAR2/GPR43 and its potential use as a novel therapeutic target in health disorders, such as obesity and insulin resistance (Tiwari, 2010; Hudson et al., 2012). FFAR2 activation has also been extensively discussed in the context of gut health. As FFAR2 is activated by SCFAs in humans, the production of these FFAs from dietary fiber fermentation in the colon has been shown to have positive effects on gut homeostasis and regulate inflammation via FFAR2 (Koh et al., 2016; Tan et al., 2017).

Although significant progress has been made in understanding the molecular signaling and physiological function of FFAR2 in humans and mice, specific functional data for the bovine receptor (bFFAR2) are scarce, mainly limited to mRNA expression and protein abundance in different tissues (Mielenz, 2017; Durand et al., 2024). Nevertheless, studies suggest that bFFAR2 may play an important role in bovine metabolism. For example, bFFAR2 has been implicated as a mediator of the regulatory effects of rumen-produced SCFAs on rumen development and insulin and glucagon secretion (Wang et al., 2009) and as a regulator of immune response through granule release from bovine neutrophils (Carretta et al., 2013). Furthermore, the already established effects of FFAR2 in reducing lipolysis observed in mice raise the hypothesis that this receptor may also be important in cattle to reduce FFA influx into the liver and maintain insulin sensitivity (Friedrichs et al., 2014).

Signaling through FFAR2, like that of any GPCR, is highly complex. GPCR signaling may be modulated by various factors, including G protein subtype, β-arrestin recruitment profile (Wootten et al., 2018), and their subcellular location, such as the plasma membrane (PM) or endocytic compartments (Eiger et al., 2022). A previous study comparing bovine and human FFAR2 receptors reported differences in ligand selectivity regarding β-arrestin 2 recruitment and G protein activity through GTPγS binding experiments (Hudson et al., 2012). Although these authors characterized bovine receptor activation and highlighted differences with the human receptor, their results were restricted to bFFAR2 upstream signaling. The distinct capacity of ligands to induce bFFAR2 conformational changes and activate the signaling pathway at different levels of the cascade remains unexplored.

In this scenario, conducting in-depth, species-specific studies on the effects of endogenous ligands on the bFFAR2 signaling pathway is an important initial step toward a better understanding of the receptor’s pharmacological properties and its potential impact on overall metabolism in bovines. The objective of the current study was to investigate the ability of a variety of FFAs to activate bFFAR2, from upstream to downstream signaling. We aimed to define the subtypes of G proteins that are recruited upon receptor stimulation; confirm the activation of the recruited G proteins and Gα_i_ subunits; characterize β-arrestin 2 recruitment, bFFAR2 internalization from the PM, and its intracellular trafficking; and evaluate the transcription of two reported genes as indicators of G proteins downstream signaling.

## 2 Materials and methods

### 2.1 Free fatty acids

Acetic acid (C2:0; Cat. No. S2889-250G), valeric acid (C5:0; Cat. No. 240370-5ML), 3-methyl-butanoic acid (3MB; Cat. No. 129542-100ML), 2-methyl-propanoic acid (2MP; Cat. No. I1754-100ML), and 2-methyl-butanoic acid (2MB; Cat. No. 193070-25G) were purchased from Sigma-Aldrich (St. Louis, United States). Propionic acid (C3:0; Cat. No. 10-0300-13), butyric acid (C4:0; Cat. No. 10-0400-13), caproic acid (C6:0; Cat. No. 10-0600-13), heptylic acid (C7:0; Cat. No. 10-0700-13), caprylic acid (C8:0; Cat. No. 10-0800-13), and capric acid (C10:0; Cat. No. 10-1000-13) were purchased from Larodan (Solna, Sweden). FFAs used for cell stimulation were diluted in either double-distilled water or absolute ethanol (VWR, Radnor, United States; Cat. No. 20821.365) according to their solubility properties and then stored at −20 °C. Prior to stimulation, FFAs were brought to room temperature and diluted in Dulbecco’s modified Eagle medium (DMEM) (no phenol red; Gibco, Waltham, United States; Cat. No. 21063029) at the appropriate concentrations.

### 2.2 Cell culture

Human embryonic kidney 293A cells (HEK293A; Invitrogen, Waltham, United States; Cat. No. 51-0036) were maintained in DMEM (Eurobio, Les Ulis, France; Cat. No. CM1DME68-01) supplemented with 10% (v/v) heat-inactivated fetal calf serum (Eurobio, Les Ulis, France; Cat. No. CVFSVF06-01) and 1% (v/v) penicillin/streptomycin mixture (Eurobio, Les Ulis, France; Cat. No. CABPES01-0U) at 37 °C in a humidified atmosphere containing 5% CO_2_. Cells were used up to the 15th passage. Cells were grown until 90%–100% visual confluency and then plated at 35,000 cells/well in a 96-well white plate (Corning, United States; Cat. No. 353296). Before adding HEK293A cells, the 96-well plates were pre-coated with 0.1% poly-L-lysine solution (Sigma-Aldrich, St. Louis, United States; Cat. No. P8920-100ML) in order to enhance cell adhesion.

### 2.3 Plasmid and biosensor constructs

A plasmid encoding *Bos taurus* FFAR2/GPR43 (bFFAR2; NM_001163784.1) was synthesized by Twist Bioscience (South San Francisco, United States). The bFFAR2 plasmid utilized for G protein recruitment, β-arrestin 2 recruitment, and internalization and trafficking experiments contained a *Renilla reniformis* luciferase (Rluc8) attached to its C terminus (Loening et al., 2006; So et al., 2006) and a FLAG epitope tag attached to its N terminus (5′-ATGGACTACAAAGACGATGACGACAAG-3′). In contrast, the bFFAR2 plasmid used for ONE vector G protein Optical (ONE-GO) biosensors, Gα_i_ activity, and diacylglycerol (DAG) production assay only contained the FLAG epitope tag attached to its N terminus. Transfections for BRET assays were performed with Metafectene PRO (Biontex, Mainz, Germany; Cat. No. T040-1.0), according to the manufacturer’s instructions for suspension cells.

G protein recruitment (Gα_s_, Gα_i_, Gα_q_, and Gα_12_) was assessed by transiently transfecting cells with the previously described bFFAR2 receptor and with plasmids encoding for mini G (mG) proteins N-terminally fused with acceptor Venus (Lan et al., 2011; Wan et al., 2018). The receptor was transfected with each mG protein individually at a 1:1 ratio (30 ng/well). For the measurement of β-arrestin 2 recruitment, cells were transfected with bFFAR2 and β-arrestin 2 (N-terminally fused with acceptor yPET) in a 2:1 ratio (30 and 15 ng/well).

For the measurement of real-time Gα_i_ activity, cells were transiently transfected with plasmids coding for bFFAR2 and BRET-based cAMP sensor CAMYEL at a 1:6 ratio (10 and 60 ng/well). Internalization and trafficking of bFFAR2 were assessed using two different sensors (LYN and FYVE). LYN and FYVE were C-terminally fused with acceptor yPET, and transfection was performed at a 1:1 ratio with bFFAR2 (30 ng/well).

We performed different activity assays using the ONE-GO biosensors (Addgene, Cambridge, United States; kit #1000000224): the Gα_i_ subunit family (Gα_i1_, Gα_i2_, Gα_i3_, Gα_OA_, Gα_OB_, and Gα_Z_), Gα_s_, and Gα_q_. Cells were transiently transfected with plasmid coding for bFFAR2 and ONE-GO biosensors at a 1:1 ratio (30 ng/well). Control assays were also performed for the Gα_q_ and Gα_i_ subunit family, in which HEK293A cells were transfected with ONE-GO sensors alone (30 ng/well), without bFFAR2. ONE-GO biosensors consist of a Gα subunit tagged with YFP and a NanoLuc luciferase detector module that recognizes GTP-bound Gα (Janicot et al., 2024). The BRET signal increases when there is Gα–GTP formation, indicating GPCR activity. Endogenous Gα_q_ activity was assessed by measuring DAG production at the PM by enhanced bystander BRET (ebBRET). Cells were transiently transfected with plasmids coding for bFFAR2 (30 ng/well), the DAG ebBRET sensor (Rluc_C1B) (3 ng/well), and the PM sensor rGFP_CAAX (30 ng/well).

Plasmids of mG proteins were a kind gift from Dr. Nevin A. Lambert from Augusta University (Augusta, Georgia, United States), while the ONE-GO biosensor kit (Addgene kit #1000000224) was a kind gift from Dr. Mikel Garcia-Marcos from Boston University (Boston, Massachusetts, United States). Plasmids encoding for β-arrestin 2, CAMYEL, LYN-YpET, and FYVE-YpET were provided by Dr. Frédéric Jean-Alphonse and Dr. Eric Reiter at INRAE (Nouzilly, France) (De Pascali et al., 2021). Plasmids encoding for DAG (Rluc_C1B) and rGFP_CAAX were a kind gift from Dr. Michel Bouvier at Montreal University (Namkung et al., 2016; Wright et al., 2021). All plasmid quantities were established as the best ratio/amounts for BRET analysis following systematic comparisons. The sequences of all constructs were verified by Sanger sequencing at Azenta Life Sciences (Griesheim, Germany).

### 2.4 Cell stimulation and BRET measurement

Forty-eight hours after cell transfection, the medium was replaced with no phenol red DMEM at 37 °C for 15 min. The medium was replaced to avoid the stimulation of the receptors by FFAs that might be present in the medium containing FBS. After 15 min, a baseline measurement was obtained by removing the medium and adding the luciferase substrate diluted in DMEM, without any ligands present. Signals were recorded for 5 min using a multimode microplate reader (VICTOR Nivo, PerkinElmer, Waltham, United States). For G protein and β-arrestin 2 recruitment, internalization and trafficking, and Gα_i_ activity assays, the luciferase substrate used was coelenterazine H (Interchim, Montluçon, France; Cat. No. FP-R3078C, final dilution 5 µM), and the emission filters were 480/30 and 530/30 nm. For the ONE-GO biosensor assays, the luciferase substrate used was Nano-Glo (Promega, Madison, United States; Cat. No. N1120, final dilution 1:200), and the emission filters were 460/40 and 535/15 nm. For DAG measurement, the luciferase substrate used was Prolume Purple (Nanolight Technology; Cat. No. 369, final dilution 2.5 µM), and the emission filters were 535/15 and 410/80 nm. After baseline measurement, the medium was removed, and then ligands and the luciferase substrate were added to the cells. Signals were recorded immediately for 10 min (G protein and β-arrestin 2 recruitment), 57 min (internalization and trafficking), 40 min (ONE-GO biosensors assays), or 5 min 30 s (DAG measurement), following the same emission filter parameters described above.

In the case of the Gα_i_ activity assay, however, after baseline measurement, cells were incubated with DMEM containing 5 µM of coelenterazine H and 10 µM of forskolin (MP Biomedicals, Santa Ana, United States; Cat. No. 11446071). Signals were recorded for 5 min using a multimode microplate reader. Finally, cells were incubated with DMEM containing 5 µM of coelenterazine H, 10 µM of forskolin, and ligands at determined concentrations. Signals were recorded immediately during 40 min following the same emission filter parameters described above.

All the assays contained control wells, in which cells were stimulated with the respective FFA solvent and luciferase substrate diluted in DMEM. For the cells incubated with FFAs diluted in absolute ethanol, the solvent was present at a maximum 1% concentration (v/v) in the final stimulation solution. Since acetic acid was obtained by the hydrolysis of sodium acetate, in the assay with this ligand, control wells also contained NaCl (Sigma-Aldrich, St. Louis, United States; Cat. No. S9888-25G). For the cAMP production assay, controls also contained forskolin at 10 µM and DMSO at 0.6%.

The net BRET signals were calculated by subtracting the ratio emission 2/emission 1 of cells stimulated with ligands from the same ratio of control cells, all multiplied by a constant 10^3^. The results (net BRET or area under the curve) are shown as the mean ± SD from at least three independent experiments carried out in triplicate.

### 2.5 Luciferase reporter assays

HEK293A cells were transiently transfected with bFFAR2 and the pGL4.33(luc2P/SRE/Hygro) or pGL4.30(luc2P/NFAT-RE/Hygro) vectors (Promega; Madison, United States; Cat. No. E134A and E848A). These vectors contain response elements that drive transcription of the firefly luciferase reporter gene *luc2P* in response to the activation of MAPK/ERK (serum response element; SRE) and calcium/calcineurin signaling pathways (nuclear factor of activated T-cell response element; NFAT-RE).

bFFAR2 and SRE vectors were transfected at a 1:10 ratio (10 and 100 ng/well), while for NFAT-RE, the receptor and vector were transfected at a 1:5 ratio (10 and 50 ng/well). Transfections were performed using Metafectene Pro (Biontex, Mainz, Germany; Cat. No. T040-1.0), according to the manufacturer’s instructions for suspension cells. The medium was replaced with no phenol red DMEM overnight at 37 °C, and after 48 h of transfection, the cells were stimulated with increasing doses of FFAs for 6 h.

Luciferase activity was measured using the Bright-Glo Luciferase Assay System (Promega, Madison, United States; Cat. No. E2610), and luminescence was measured using a multimode microplate reader (VICTOR Nivo, PerkinElmer, Waltham, United States). Luminescence was calculated as the fold change over the luciferase control. The results are shown as the mean ± SD from at least three independent experiments conducted in triplicate.

### 2.6 Statistical analysis

#### 2.6.1 Statistical analysis of concentration–response curves

For G protein and β-arrestin 2 recruitment and luciferase reporter assays, concentration-response curves were fitted to the data using the “log (agonist) vs. response (three parameters)” dose-response stimulation equation in Prism Version 6.0 (San Diego, United States), which is as follows:

$$Y=Bottom+\left(Top-Bottom\right)/\left(1+10^{\left(\left(LogEC50-X\right)\right)}\right).$$

For normalization, the values are represented as the percentage of caproic acid response (% of C6:0). Efficacy (Emax) and potency (positive logarithm of the ligand EC_50_) were extracted from concentration-response curves. Data were analyzed using an unpaired t-test or one-way ANOVA, as appropriate, using Prism Version 6.0 (San Diego, United States). Significance was declared at *p-value* < 0.05.

#### 2.6.2 Statistical analysis of internalization and trafficking and Gα_i_/Gα_q_ activity

The results were normalized as the percentage of caproic acid response (% of C6:0) and analyzed using an unpaired t-test or one-way ANOVA, as appropriate, using Prism Version 6.0 (San Diego, United States). Significance was declared at *p-value* < 0.05.

## 3 Results

### 3.1 Endogenous bFFAR2 ligands elicit distinct G protein and β-arrestin 2 recruitment profiles

**FIGURE 1:**
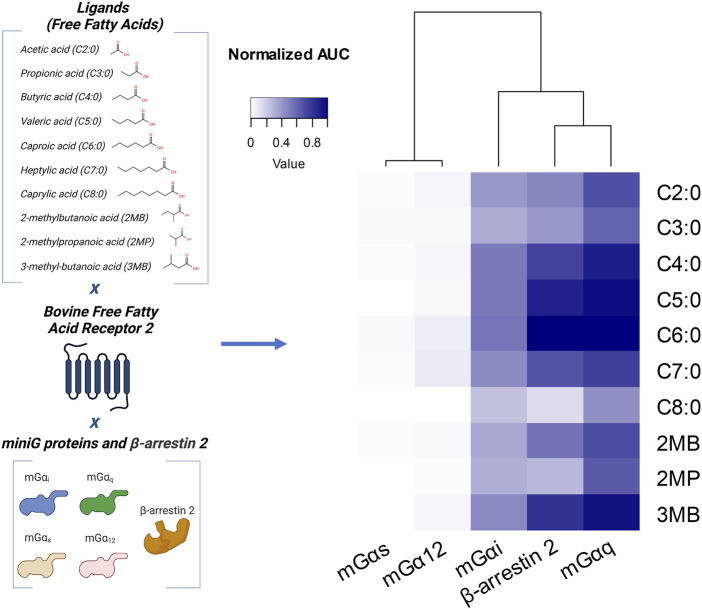
Profiling of G protein and β-arrestin 2 recruitment to bFFAR2 upon stimulation with different FFAs. The schematic representation of the variables studied is given on the left. Heatmap showing the recruitment of G proteins (mGα_i_, mGα_q_, mGα_s_, and mGα_12_) and β-arrestin 2 to bFFAR2 in HEK293A cells is given on the right. Cells were stimulated with C2:0 at 0.1 M, C8:0 at 1 mM, and the other FFAs at 3.16 mM. The doses of FFAs selected for this study were based on the plasma concentrations observed in bovines. Concentrations of FFAs applied to the HEK293A cells aimed to achieve the maximum of approximately 100-fold the physiological levels. BRET signals were monitored for 10 min after stimulation with FFAs, and AUC was generated for each ligand. The results of G proteins (mGα_s_, mGα_i_, mGα_q_, and mGα_12_) were normalized to C6:0 in the mGα_q_ recruitment assay, while results of β-arrestin 2 were normalized to C6:0 in the β-arrestin 2 recruitment assay. Results are from at least three independent experiments. Illustration was created using BioRender (https://biorender.com/).

We first assessed whether the four major families of G protein Gα subunit (Gα_s_, Gα_i_, Gα_q_, and Gα_12_) and β-arrestin 2 are recruited to bFFAR2 under stimulation by endogenous ligands (FFAs). Bioluminescence energy transfer (BRET) was used for quantitative monitoring of G protein Gα subunits or β-arrestin 2 interactions with bFFAR2. We used bFFAR2 fused to the *Renilla reniformis* luciferase (bFFAR2-Rluc8) as the donor and miniG protein biosensors NES-Venus_mG probes (Wan et al., 2018), which are further denoted as mGα_s_, mGα_i_, mGα_q_, and mGα_12_, and YPET-β-arrestin 2 (Kamal et al., 2009) were used as the acceptors. HEK293A cells expressing the different BRET partners were stimulated with different FFAs for 10 min. BRET signals generated by bFFAR2 interacting with either mGα subunits or β-arrestin 2 were recorded, and the area under the curve (AUC) was generated (Figure 1). The results of G proteins (mGα_s_, mGα_i_, mGα_q_, and mGα_12_) were normalized to C6:0 in the mGα_q_ recruitment assay, while the results of β-arrestin 2 were normalized to C6:0 in the β-arrestin 2 recruitment assay. We observed a substantial increase in the overall normalized AUC for the mGα_i_ and mGα_q_ Gα subtypes and β-arrestin 2, indicating the recruitment of these proteins to the receptor upon stimulation. In contrast, the recruitment of mGα_12_ and mGα_s_ to the receptor was not observed for any of the studied ligands. The normalized results for mGα_12_ and mGα_s_ recruitment represented only 0.01 ± 0.01 and 0.04 ± 0.02 (mean ± SD) as a ratio to the maximum AUC, respectively, and the kinetics curves of the FFA-stimulated cells were not distinguishable from those of the unstimulated control wells (Supplementary Figure S1).

**FIGURE 2:**
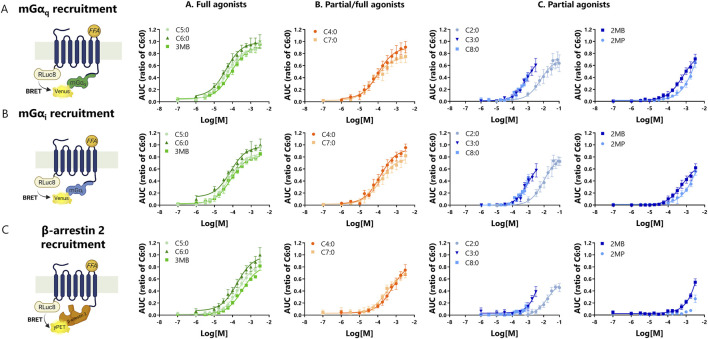
Concentration-response curves of bFFAR2 vs. FFAs in HEK293A cells. **(A)** mGα_q_ recruitment, **(B)** mGα_i_ recruitment, and **(C)** β-arrestin 2 recruitment were measured. HEK293A cells transfected with bFFAR2 and the appropriate BRET sensors were challenged with increasing doses of FFAs. Signals were monitored for 10 min. The AUC generated by each compound was plotted and fitted using the “log(agonist) vs. response (three parameters)” dose-response stimulation equation in Prism 6.0 (San Diego, CA, United States). The results for concentration-response curves are normalized as a ratio of C6:0 within each assay and are shown as the mean ± SD from at least three independent experiments. Color coding represents the classification of FFAs into three different groups based on their maximum response toward the three different recruitment assays (mGα_q_, mGα_i_, and β-arrestin 2): **(A)** full agonists (green; C5:0, C6:0, and 3MB), **(B)** partial/full agonists (orange; C4:0 and C7:0), and **(C)** partial agonists (blue; C2:0, C3:0, C8:0, 2MB, and 2MP). Illustration was created using BioRender (https://biorender.com/).

We next evaluated the pharmacological properties of FFAs for mGα_i_, mGα_q_, and β-arrestin 2 recruitment to the bFFAR2. HEK293A cells expressing the different BRET partners, as previously described, were stimulated with increasing concentrations of the different ligands over a 10-min period. Kinetics were generated for each concentration, and the AUC was plotted as concentration-response curves (Figure 2). Data are presented as the ratio of C6:0 maximal effect within each assay. C6:0 was selected as the reference as it showed the maximum response for all the signaling pathways analyzed. We observed that the FFAs presented clear differences in their pharmacological profiles in activating bFFAR2 (Table 1; Figure 2). FFAs with up to eight carbons were capable—at different intensities—of inducing bFFAR2 activation, defined in this study as the ability to recruit mGα_i_, mGα_q_, and β-arrestin 2 to the receptor, as measured by BRET analysis. The FFA C10:0 failed to induce any significant response (data not shown), indicating a cut-point in terms of carbon length for the activation of G protein and β-arrestin 2 signaling to the receptor.

**TABLE 1:** Comparison of potencies (pEC_50_) and efficacies (Emax) of FFAs to recruit mGα_q_, mGα_i_, and β-arrestin 2 to bFFAR2 after 10 min of stimulation. Potency values were represented as pEC_50_ ± SD and analyzed using a one-way ANOVA using Prism Version 6.0 (San Diego, CA, United States), in which mean values with different superscripts were significantly different after Tukey’s correction for multiple comparisons (*p-values* < 0.05). Efficacy values were represented as a ratio over C6:0 Emax ± SD and analyzed in comparison with C6:0 using an unpaired t-test using Prism Version 6.0 (San Diego, CA, United States). * *p-values* < 0.05; *** *p-values* < 0.001.

Ligand	mGα_i_ recruitment	mGα_q_ recruitment	β-arrestin 2 recruitment
pEC_50_ ± SD			
C5:0	4.21 ± 0.09^a^	4.32 ± 0.07^a^	3.64 ± 0.07^ab^
C6:0	4.14 ± 0.13^a^	4.35 ± 0.11^a^	3.75 ± 0.13^a^
3MB^3^	4.12 ± 0.07^a^	4.09 ± 0.07^a^	3.46 ± 0.07^ab^
C4:0	3.89 ± 0.10^a^	3.99 ± 0.13^a^	3.20 ± 0.15^ac^
C7:0	3.90 ± 0.13^a^	4.15 ± 0.13^a^	3.52 ± 0.11^ab^
C2:0	2.01 ± 0.15^d^	2.17 ± 0.19^c^	1.75 ± 0.16^c^
C3:0	3.01 ± 0.19^bc^	3.05 ± 0.17^b^	1.97 ± 0.90^bc^
C8:0	2.57 ± 0.40^c^	2.96 ± 0.20^b^	2.55 ± 1.28^ac^
2MB^1^	3.21 ± 0.11^b^	3.17 ± 0.10^b^	1.68 ± 0.59^c^
2MP^2^	ND	ND	NF
Emax (ratio over C6:0) ± SD			
C5:0	0.96 ± 0.02	0.97 ± 0.04	0.89 ± 0.01
C6:0	1.00 ± 0.10	1.00 ± 0.12	1.00 ± 0.12
3MB^3^	0.85 ± 0.03	0.96 ± 0.06	0.82 ± 0.07
C4:0	0.96 ± 0.06	0.91 ± 0.10	0.75 ± 0.09*
C7:0	0.82 ± 0.12	0.75 ± 0.10*	0.68 ± 0.06*
C2:0	0.74 ± 0.10*	0.70 ± 0.13*	0.47 ± 0.04***
C3:0	0.58 ± 0.11*	0.61 ± 0.11*	0.40 ± 0.10***
C8:0	0.44 ± 0.07*	0.44 ± 0.04*	0.13 ± 0.07***
2MB^1^	0.62 ± 0.07*	0.71 ± 0.08*	0.55 ± 0.06*
2MP^2^	ND	ND	NF

^1^2MB, 2-methyl-butanoic acid; ^2^2MP, 2-methyl-propanoic acid; ^3^3MB, 3-methyl-butanoic acid; ND, not determined; NF, values do not fit the concentration-response curve.

Furthermore, since the concentration-response curves for neither mGα_i_, mGα_q_, nor β-arrestin 2 recruitment upon 2MP stimulation reached the expected plateau (Figure 2), the pharmacological characterization of this ligand was carefully considered. For β-arrestin 2, the recruitment results were weak compared to those of C6:0 and did not fit the concentration-response equation (ambiguous fitting). In this regard, 2MP was considered to have little pharmacological activity toward β-arrestin 2 under our experimental conditions, and the pharmacological parameters are shown as NF (not fitting) in Table 1. For mGα_i_ and mGα_q_ recruitment upon 2MP stimulation, responses were moderate compared to those of C6:0, and data fit the concentration-response equation; however, considering the curves’ shapes, the potencies and efficacies were not considered in our general comparative analyses and are shown as ND (not determined) in Table 1.

FFAs were classified as full or partial agonists based on their maximum response (efficacy) in comparison with the reference ligand (C6:0) (Table 1). Along with C6:0, C5:0 and 3MB (an isomer of C5:0) behaved as full agonists in the mGα_i_, mGα_q_, and β-arrestin 2 recruitment assays. The FFA C4:0 was also considered a full agonist for the recruitment of mGα_i_ and mGα_q_ but a partial agonist for β-arrestin 2. C7:0 was a full agonist for mGα_i_ recruitment but a partial agonist for mGα_q_ and β-arrestin 2. C2:0, C3:0, C8:0, and 2MB behaved as partial agonists for mGα_i_, mGα_q_, and β-arrestin 2 recruitment assays. Although efficacy values were not extracted from the concentration-response curves upon 2MP stimulation, this FFA was considered an exceptionally weak partial agonist in these assays. Based on their efficacies toward G protein and β-arrestin signaling pathways, we classified the studied FFAs into the following groups: A. full agonists (C5:0, C6:0, and 3MB), B. partial/full agonists (C4:0 and C7:0), and C. partial agonists (C2:0, C3:0, C8:0, 2MB, and 2MP).

**FIGURE 3:**
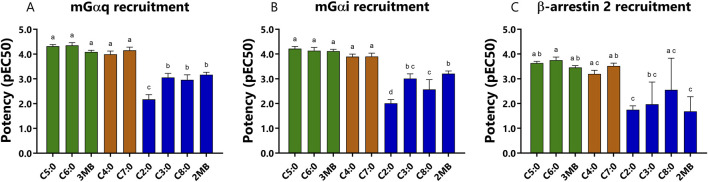
Comparison of potencies (pEC_50_) of each FFAs to recruit **(A)** mGα_q_, **(B)** mGα_i_, and **(C)** β-arrestin 2 to bFFAR2 after 10 min of stimulation. Potency values were represented as the positive logarithm of the ligand EC_50_ concentration. Results were analyzed using a one-way ANOVA using Prism Version 6.0 (San Diego, CA, United States). The results are shown as the mean ± SD from at least three independent experiments. The mean values with different superscripts were significantly different after Tukey’s correction for multiple comparisons (*p-values* < 0.05). Color coding represents the classification of FFAs into three different groups based on their maximum response toward the three different recruitment assays (mGα_q_, mGα_i_, and β-arrestin 2): **(A)** full agonists (green; C5:0, C6:0, and 3MB), **(B)** partial/full agonists (orange; C4:0 and C7:0), and **(C)** partial agonists (blue; C2:0, C3:0, C8:0, and 2MB).

The study of potencies by which FFAs activated bFFAR2 mGα_i_, mGα_q_, and β-arrestin 2 signaling reveals a distinct rank order (Table 1; Figure 3). For mGα_i_ and mGα_q_ recruitment, potencies increased with FFAs of longer chain lengths, up to seven carbons, such that C4:0 = C5:0 = C6:0 = C7:0 > C3:0 > C2:0 (Figures 3A, B). Beyond C7:0, the addition of one carbon (C8:0) significantly reduced the potency for mGα_i_ and mGα_q_ recruitment. Interestingly, 2MB and 3MB presented a different pattern of potencies. While 3MB, an isomer of C5:0, showed full potencies, 2MB, another isomer of C5:0, showed reduced potencies for mGα_i_ and mGα_q_ recruitment compared with C5:0 and 3MB. For β-arrestin 2 recruitment, C6:0 was the most potent, although FFAs with four to seven carbons plus 3MB presented similar potencies (Figure 3C). Although C8:0 and C6:0 were statistically equally potent, the concentration-dose responses (Figure 2) show a clear difference between these ligands in recruiting β-arrestin 2 to the receptor; thus, potency values should be cautiously interpreted. Furthermore, shorter FFAs (C2:0 and C3:0) and 2MB showed the lowest potencies in recruiting β-arrestin 2 to bFFAR2 (Figure 3C).

### 3.2 Gα_i_ and Gα_q_ recruited to the bFFAR2 are subsequently activated

**FIGURE 4:**
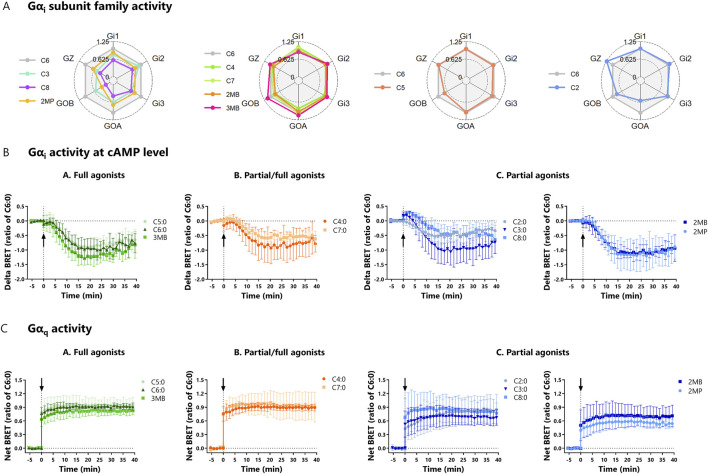
**(A)** Radar plots of Gα_i_ subunit family activity upon FFA stimulation (C2:0 at 0.1 M, C8:0 at 1 mM, and the other FFAs at 3.16 mM). HEK293A cells were transfected with bFFAR2 and ONE-GO biosensors (Gα_i1_, Gα_i2_, Gα_i3_, Gα_OA_, Gα_OB_, and Gα_Z_, individually) at a 1:1 ratio. FFAs were added, and signals were monitored for 40 min. The AUC generated by each compound was plotted as the ratio of C6:0 in radar plots. The results are shown as the mean from at least three independent experiments. **(B)** Kinetic curves of Gα_i_ activity measured by cAMP production upon FFA stimulation (C2:0 at 0.1 M and other FFAs at 3.16 mM). HEK293A cells were transfected with bFFAR2 and cAMP sensor CAMYEL at a 1:6 ratio. Cells were first stimulated with forskolin, which is a direct activator of adenylyl cyclase that results in higher levels of cAMP in the cell. Then, FFAs were added (time = 0 min), and signals were monitored for 40 min. As FFAs activate Gα_i_, an inhibitor of adenylyl cyclase, there is a decrease in cellular cAMP. Delta BRET was calculated as the difference in net BRET of cells stimulated with FFA + forskolin and cells stimulated with forskolin only. **(C)** Kinetic curves of Gα_q_ activity upon FFA stimulation (C2:0 at 0.1 M and other FFAs at 3.16 mM). HEK293A cells were transfected with bFFAR2 and ONE-GO biosensor at a 1:1 ratio. Then, FFAs were added (time = 0 min), and signals were monitored for 40 min. Gα_q_ activity was measured by a Gα_q_–GTP detector molecule. Kinetic curves were plotted as a function of time. The results are shown as the mean ± SD from at least three independent experiments. Color coding represents the classification FFAs into three different groups based on their maximum response toward the three different recruitment assays (mGα_q_, mGα_i_, and β-arrestin 2): **(A)** full agonists (green; C5:0, C6:0, and 3MB), **(B)** partial/full agonists (orange; C4:0 and C7:0), and **(C)** partial agonists (blue; C2:0, C3:0, C8:0, 2MB, and 2MP).

After establishing that mGα_i_ is recruited to bFFAR2 upon FFA stimulation, we investigated the capacity of the selected ligands to activate the specific members of the Gα_i_ subunit family, namely, Gα_i1_, Gα_i2_, Gα_i3_, Gα_OA_, Gα_OB_, and Gα_Z_. Overall, we showed that all FFAs induced, with different intensities, the formation of Gα–GTP with the six members of the Gα_i_ subunit family (Figure 4A). We observed that C4:0, C7:0, 2MB, and 3MB exhibited a pattern of activation similar to that of the reference ligand C6:0. In contrast, 3MB and C2:0 induced a substantial activity through Gα_OB_ and Gα_z_, respectively, compared to C6:0, while C3:0, C8:0, and 2MP poorly activated Gα_OB_. Control assays performed with cells not expressing the receptor confirm the bFFAR2-dependent activity of the Gα_i_ subunit family (Supplementary Figure S2).

We next studied whether ligand-induced bFFAR2 coupling to Gα_i_ resulted in the activation of this protein at the cAMP level. Gα_i_ activation has been reported to decrease cAMP production in cells. We, therefore, tested the ability of bFFAR2-recruited Gα_i_ to decrease forskolin-induced cAMP production. cAMP production was monitored using the previously reported cytoplasmic BRET sensor CAMYEL (Jiang et al., 2007). A delta BRET was calculated by subtracting the BRET signal in cells stimulated with forskolin only from the BRET signal in cells stimulated with FFA + forskolin. Our results confirm the expected decrease in delta BRET, indicating a reduction in forskolin-induced cellular cAMP production upon FFA stimulation (Figure 4B). As a percentage of forskolin response (100%), results for the FFA-stimulated cells ranged from 55% ± 7% (2MP) to 83% ± 16% (C7:0). Overall, stimulation with all the studied FFAs reduced (*p-value* = 0.02) cAMP production in HEK293A cells compared with forskolin alone, indicating the activation of Gα_i_ signaling. However, no differences (*p-value* > 0.05) were observed between FFAs in their capacity to reduce cAMP production. In addition, using the ONE-GO Gα_s_ biosensor to detect the Gα_s_–GTP active form, we investigated whether the initial delta BRET increase observed in the kinetic curves of some FFAs (C3:0 and C8:0, for example) (Figure 4B) was related to Gα_s_ activity (cAMP production). Overall, results show the activation of Gα_s_ in HEK293A cells (Supplementary Figure S3A), although in relatively low amounts compared to Gα_i2_ activity (Supplementary Figure S3B). The fact that BRET signals generated by ONE-GO biosensors are not directly linked to bFFAR2 activation, combined with the inability of bFFAR2 to couple to Gα_s_, as evidenced by the recruitment assay with mGα_s_ (Supplementary Figure S1), suggests that increases in delta BRET observed in the Gα_i_ activity assay (Figure 4B) might be unrelated to bFFAR2 and instead result from the activation of other GPCRs endogenously expressed in HEK293A cells.

In terms of Gα_q_, we investigated its activity using the Gα_q_ ONE-GO biosensor (Janicot et al., 2024). The kinetic curves show a rapid increase in net BRET, demonstrating the formation of Gα_q_–GTP upon ligand stimulation (Figure 4C). Overall, all the studied FFAs induced Gα_q_–GTP formation (*p-value* < 0.01), evidencing that stimulation of bFFAR2 results in the activation of Gα_q_. However, no differences (*p-value* > 0.05) were observed between FFAs in their capacity to induce Gα_q_–GTP formation. Furthermore, by comparing the results from cells expressing bFFAR2 with those that did not, we observed that the bFFAR2-dependent Gα_q_ activity was greater than 60% for all the ligands tested (83% ± 9.1%) (Supplementary Figure S4). bFFAR2 signaling was associated with more than 90% of the total Gα_q_ activity when cells were stimulated with C6:0, 3MB, and C7:0. bFFAR2 endogenous Gα_q_ activation was evaluated using the DAG ebBRET biosensor (Rluc_C1B) and rGFP_CAAX construct. The kinetic curves (Supplementary Figure S5A) and the linked positive AUC (Supplementary Figure S5B) confirm that bFFAR2 triggers DAG production at the PM for all the tested ligands, thus further confirming Gα_q_ activation.

### 3.3 Gα_i_ and Gα_q_ recruited to the bFFAR2 induce nuclear gene expression

We provided evidence of bFFAR2 coupling to both Gα_i_ and Gα_q_ using the BRET assay, and in order to comprehend the downstream effects of these signaling pathways, a luciferase reporter assay was used, assessing two different response elements. Gene transcription of the SRE was measured as an indication of downstream signaling through Gα_i_, while the transcription of the NFAT-RE was used as evidence of downstream signaling through Gα_q._ Upon receptor activation, Gα_i_ dissociates from the βγ subunit, which then activates the MAP kinase pathway and induces SRE transcription. Meanwhile, the activation of the Gα_q_ pathway activates phospholipase C, resulting in increases in inositol 1,4,5-triphosphate (IP3) and DAG, leading to the mobilization of calcium from intracellular stores, which, in turn, activates NFAT-RE.

**FIGURE 5:**
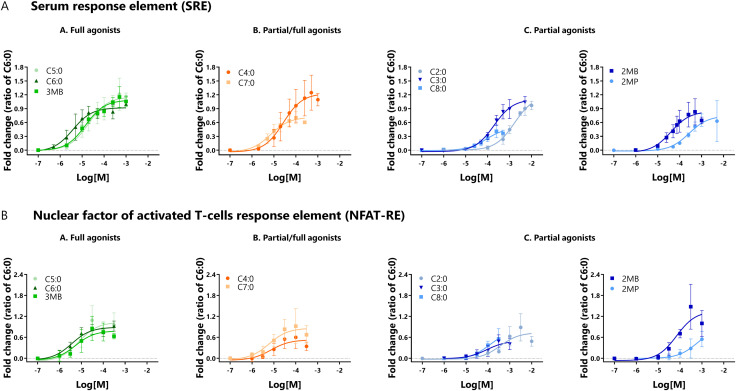
Activity of bFFAR2 vs. FFAs on downstream signaling, which was assessed by the luciferase reporter gene assay. HEK293A cells co-expressing bFFAR2 and **(A)** SRE or **(B)** NFAT-RE were stimulated with increasing concentrations of FFAs. Gene transcription of SRE and NFAT-RE were measured as indicators of downstream signaling through Gα_i_ and Gα_q_, respectively. Luciferase activities were recorder 6 h after stimulation. Luminescence was calculated as fold change over the luciferase control and plotted using the“log (agonist) vs. response (three parameters)” dose-response stimulation equation in Prism 6.0 (San Diego, CA, United States). Results are shown as the ratio of C6:0 within each assay and presented as the mean ± SD from at least three independent experiments. Color coding represents the classification of FFAs into three different groups based on their maximum response toward the three different recruitment assays (mGα_q_, mGα_i_, and β-arrestin 2): **(A)** full agonists (green; C5:0, C6:0, and 3MB), **(B)** partial/full agonists (orange; C4:0 and C7:0), and **(C)** partial agonists (blue; C2:0, C3:0, C8:0, 2MB, and 2MP).

HEK293A cells co-expressing the bFFAR2 and the luciferase reporter genes were stimulated with increasing concentrations of FFAs over a 6-h period. Luminescence results were calculated as fold change over the luciferase control and plotted as concentration-response curves (Figure 5). Data are presented as the ratio of C6:0. Efficacies and potencies of FFAs to induce SRE and NFAT-RE gene transcription are presented in Table 2. Compared with the reference compound C6:0, we observed that C7:0 and C8:0 behaved as partial agonists for the SRE assay, and C3:0, C4:0, C8:0, and 2MP were classified as partial agonists for the NFAT-RE assay. The other FFAs studied behaved as full agonists for both assays (Table 2). The greatest potency for the SRE assay was observed for C6:0, followed by C7:0 and 3MB. In contrast, C2:0 and 2MP showed the lowest potencies in inducing SRE transcription (Table 2). The ranking of potencies of the studied FFAs to induce NFAT-RE transcription was similar to that observed for the SRE assay.

**TABLE 2:** Comparison of potencies (pEC_50_) and efficacies (Emax) of FFAs to induce gene transcription by SRE and NFAT-RE. End-point luminescence values were observed after 6 h of stimulation. Potency values were represented as pEC_50_ ± SD and analyzed using a one-way ANOVA using Prism Version 6.0 (San Diego, CA, United States), in which mean values with different superscripts were significantly different after Tukey’s correction for multiple comparisons (*p-values* < 0.05). Efficacy values were represented as a ratio over C6:0 Emax ± SD and analyzed in comparison with C6:0 using an unpaired t-test using Prism Version 6.0 (San Diego, CA, United States). * *p-values* < 0.05; *** *p-values* < 0.001.

Ligand	SRE	NFAT-RE
pEC_50_ ± SD		
C5:0	4.72 ± 0.36^ad^	5.12 ± 0.61^ab^
C6:0	5.44 ± 0.35^a^	5.37 ± 0.49^a^
3MB^3^	4.83 ± 0.26^abc^	5.21 ± 0.55^ab^
C4:0	4.58 ± 0.41^ade^	5.08 ± 0.52^ab^
C7:0	5.18 ± 0.37^ab^	5.27 ± 0.56^ab^
C2:0	2.81 ± 0.27^f^	3.44 ± 0.52^c^
C3:0	3.73 ± 0.22^df^	4.01 ± 0.59^bc^
C8:0	4.10 ± 0.40^cde^	4.05 ± 0.63^bc^
2MB^1^	4.42 ± 0.44^bde^	4.20 ± 0.43^ac^
2MP^2^	3.63 ± 0.28^ef^	2.96 ± 0.93^c^
Emax (ratio over C6:0) ± SD		
C5:0	1.30 ± 0.27	1.15 ± 0.40
C6:0	1.00 ± 0.00	1.00 ± 0.00
3MB^3^	1.18 ± 0.19	0.90 ± 0.31
C4:0	1.27 ± 0.34	0.65 ± 0.30*
C7:0	0.71 ± 0.12*	0.95 ± 0.47
C2:0	0.99 ± 0.07	0.89 ± 0.39
C3:0	0.98 ± 0.24	0.50 ± 0.18***
C8:0	0.43 ± 0.13*	0.55 ± 0.30*
2MB^1^	0.83 ± 0.28	1.48 ± 0.64
2MP^2^	0.72 ± 0.22	0.55 ± 0.22*

^1^2MB, 2-methyl-butanoic acid; ^2^2MP, 2-methyl-propanoic acid; ^3^3MB, 3-methyl-butanoic acid.

### 3.4 Not all bFFAR2 ligands target the receptor in the same internalization compartments

**FIGURE 6:**
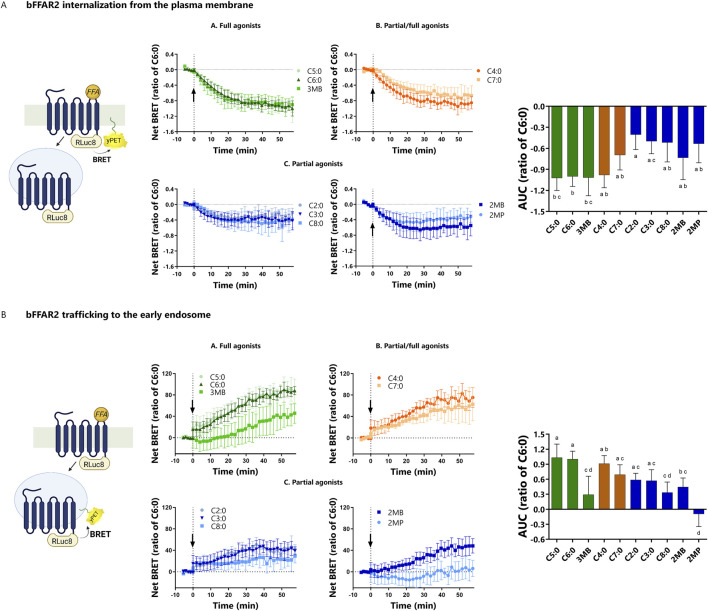
bFFAR2 internalization and trafficking upon FFA stimulation (C2:0 at 0.1 M and other FFAs at 3.16 mM). HEK293A cells were transfected with bFFAR2 and LYN or FYVE BRET sensors at a 1:1 ratio. Following FFA stimulation, signals were monitored for 57 min. The AUC was calculated from net BRET results and normalized as the ratio of C6:0 within each assay. **(A)** The LYN sensor measures the internalization of the receptor from the plasma membrane (reduction of BRET upon internalization). **(B)** FYVE is used for the trafficking of the receptor to the EE (increased BRET upon trafficking to EE). The results are shown as the mean ± SD from at least three independent experiments. Kinetic curves were plotted as a function of time. AUC were analyzed using one-way ANOVA using Prism Version 6.0 (San Diego, CA, United States). The mean values with different superscripts were significantly different after Tukey’s correction for multiple comparisons (*p-values* < 0.05). Color coding represents the classification of FFAs into three different groups based on their maximum response toward the three different recruitment assays (mGα_q_, mGα_i_, and β-arrestin 2): **(A)** full agonists (green; C5:0, C6:0, and 3MB), **(B)** partial/full agonists (orange; C4:0 and C7:0), and **(C)** partial agonists (blue; C2:0, C3:0, C8:0, 2MB, and 2MP). Illustration was created using BioRender (https://biorender.com/).

We assessed the internalization and trafficking of bFFAR2 upon FFA stimulation using two previously published BRET sensors (LYN and FYVE) (Lan et al., 2011). LYN is located in the PM, and the internalization of the receptor from the PM results in the reduction of BRET signals between the receptor and LYN (Figure 6A). Meanwhile, FYVE is selectively localized in the early endosome (EE), and as the receptors move from the PM to the EE upon agonist stimulation, there is an increase in the BRET signal (Figure 6B).

FFA stimulation resulted in the internalization of the receptor from the PM (*p-value* < 0.01), as observed by decreased BRET in the LYN assay for all the studied FFAs, which are shown as negative AUC values in this study (Figure 6A). The internalization kinetics of bFFAR2 were somewhat similar for all the ligands. Overall, the degree of bFFAR2 internalization was consistent with the classification of the ligands as partial or full agonists. The greatest internalization from the PM was observed for the FFAs of the full agonists group, while lower internalization was observed for the partial agonist group (Figure 6A).

There was an effect of FFA stimulation in the trafficking of bFFAR2 to the EE (*p-value* < 0.01), as observed by an increase in BRET in the FYVE assay (Figure 6B), which is shown as positive AUC value in this study. Overall, the trafficking of bFFAR2 to EE is consistent with internalization from the PM for all ligands. However, we reported discrepancies for both 2MP and 3MB. While 3MB induced a significant internalization of the receptor from the PM, we observed low trafficking of bFFAR2 to EE compared to that of other ligands that similarly induced receptor internalization (C5:0 and C6:0). Furthermore, 3MB exhibited a markedly slower rate of accumulation of bFFAR2 within the EE compartment. 2MP displayed an even more pronounced contrast in behavior with regard to bFFAR2 trafficking. Although 2MP induced internalization of bFFAR2 from the PM, no significant accumulation of bFFAR2 within the EE compartment was observed upon stimulation (Figure 6B).

## 4 Discussion

The ability of GPCRs to adopt a wide variety of active conformations is an important feature that enables a single GPCR to coordinate a tailored cellular response for each of its ligands. In this study, we simultaneously investigate the behavior of a wide variety of endogenous bFFAR2 ligands on (i) the recruitment of different subtypes of G proteins and β-arrestin 2; (ii) the activity of the recruited G proteins; (iii) receptor internalization from the PM and trafficking to EE; and (iv) regulation of SRE and NFAT-RE transcription as indicators of G protein downstream signaling. The pharmacological properties of each ligand, i.e., efficacy and potency for G protein and β-arrestin 2 recruitment and transcription of reporter genes, were also determined in this study. We were able to show that the different ligands displayed different behaviors at the level of bFFAR2, both in terms of signaling and internalization.

Numerous studies have demonstrated that bFFAR2 has the capacity to couple to and activate both Gα_i_ and Gα_q_ proteins under SCFA stimulation. These findings were derived from studies conducted on a bovine mammary epithelial cell line (Yonezawa et al., 2009), bovine neutrophils (Carretta et al., 2013), or bovine adipose tissue explants (Westbrook et al., 2021). Although these studies have been conducted in a cellular context where bFFAR2 is endogenously expressed, they are limited to the primary SCFAs, C2:0 and C3:0. Additionally, Gα_i_ activation upon C2:0, C3:0, and C4:0 stimulation has been demonstrated in a CHO cell line heterogeneously expressing bFFAR2 (Wang et al., 2009). In our study, we used HEK293A cells, a sub-clone of HEK293 cells. HEK293A cells are a valuable asset for studying bFFAR2 since neither FFAR2 nor FFAR3, another GPCR known to be activated by SCFAs, is endogenously expressed by these cells (Atwood et al., 2011).

A previous study (Hudson et al., 2012), conducted in a context similar to ours—namely, HEK293 cells heterogeneously expressing bFFAR2—demonstrated that bFFAR2 can couple to β-arrestin 2 following FFA binding. In this study, the authors tested a vast array of ligands, encompassing both natural and synthetic compounds. This included all the ligands tested in our study, with the exception of 2MP. Additionally, the authors confirmed bFFAR2-induced G protein activity through a GTPγS experiment. However, the specific G protein subtypes involved remained undetermined. In the present study, we directly showed that bFFAR2 couples with Gα_i_ and Gα_q_ and not with Gα_12_ and Gα_s_, which is in agreement with what is observed in human and mouse FFAR2, as reviewed by Stoddart et al. (2008).

Having demonstrated the coupling of bFFAR2 to Gα_i_, we aimed to further investigate Gα_i_ signaling by studying the capacity of FFAs to induce the activation (Gα–GTP formation) of different members of this subunit family at the receptor. The Gα_i_ subunit family has the largest number of individual members, and these members have been shown to have both overlapping and distinct functions by inducing various cellular signaling processes (Ono et al., 2023; Wettschureck and Offermanns, 2005; Villaseca et al., 2022). For example, while Gα_o_ was found to mediate the inhibition of calcium entry, Gα_i2_ inhibited cAMP accumulation in GH4C1 cells (Liu et al., 1994). Moreover, it has been demonstrated that forskolin-induced adenylyl cyclase is not inhibited to the same extent by all Gα_i_ subunits (Ghahremani et al., 1999) and that subunits behave biochemically differently from one another in terms of GDP dissociation rates (Linder et al., 1990). Overall, our results showed that our selection of endogenous ligands (C2:0 to C8:0 and branched FFAs) activated all six members of the Gα_i_ subunit family. However, we observed that each FFA presented an individual pattern of activation compared to that of the reference ligand C6:0. This information is potentially relevant to the cell biological outcomes when considering that differences between members of the Gα_i_ subunit family allow for diverse Gα_i_-coupled GPCR signal transduction (Ono et al., 2023).

Pharmacological studies of human FFAR2 have ranked C2:0, C3:0, and C4:0 as the most potent ligands for various readouts, i.e., cAMP and Ca^2+^ production, MAP kinase ERK phosphorylation, and β-arrestin 2 recruitment, with little or no effect for FFAs of six or more carbons (Brown et al., 2003; Le Poul et al., 2003; Nilsson et al., 2003; Hudson et al., 2012). Preliminary results indicated that the rank order of potency for bFFAR2 was somewhat shifted, with FFAs of four to seven carbons having the greatest potency for β-arrestin 2 recruitment and MAP kinase ERK phosphorylation (Hudson et al., 2012). By examining both upstream (G protein and β-arrestin 2 recruitment) and downstream signaling (G protein-dependent transcriptional regulation), our study provides clear confirmation that FFAs of four to seven carbon lengths are more potent in activating bFFAR2. These contrasting responses between the human and bovine receptors may be related to the fact that although GPCR orthologs from different mammalian species are assumed to be activated by the same endogenous ligands, the potency and affinity of these ligands can vary according to the physiological adaptations to factors such as diet and associated microbial challenges (Hudson et al., 2013; Schulze et al., 2022). The differences in response may be greater when considering GPCRs, where the receptors are exposed to extremely different ligand concentrations between species (Hudson et al., 2012), which may be the case for FFAR2.

In ruminants, SCFAs play an important role in the overall metabolism, accounting for approximately 70% of the total metabolizable energy (Bergman, 1990; Moran, 2005). The SCFAs, mainly C2:0, followed by C3:0 and C4:0, are largely produced (mM range) (Resende Júnior et al., 2006; McKnight et al., 2019) through the fermentation of dietary carbohydrates by the rumen microbiota and absorbed by the ruminal epithelium, reaching the liver via the portal vein (Hocquette and Bauchart, 1999). In this context, as proposed by Hudson et al. (2012), the particular pharmacology of bFFAR2, i.e., the lower potency for shorter-chain FFAs (C2:0 and C3:0) and responsiveness to longer-chain FFAs (six carbons and more), may be an evolutionary adaptation to maintain dynamic receptor function in the rumen, where bFFAR2 is known to be expressed (Wang et al., 2009; Wang et al., 2012) and where its ligands, SCFAs, are present in high concentrations. Furthermore, at the systemic level, C2:0 is present at higher concentrations in cattle, with values that can exceed 1 mM (Friedrichs et al., 2016; Li et al., 2022), than in humans, where blood concentrations are approximately 100 µM (Cummings et al., 1987; Barman et al., 2024), which may sustain a lower sensitivity of bFFAR2 to SCFAs of two to four carbon length.

Interestingly, the molecular rationale for the unique pharmacology of bFFAR2 was previously demonstrated (Hudson et al., 2012). The authors aligned the bovine and human receptors, and by selective mutagenesis of key amino acids in the binding pocket, they were able to identify a single cysteine–glycine variation that is central to the receptor’s responsiveness to longer FFAs (C5:0 to C8:0). This variation in the bFFAR2 binding pocket is present in all members of the suborder *Ruminantia*, suggesting that FFAR2 from species such as goats and sheep may share the chain-length selectivity with bFFAR2 (Hudson et al., 2013).

BSCFAs, like SCFAs with straight chains, are produced by the ruminal microbiota and are derived from the degradation of branched-chain amino acids rather than carbohydrates (Peirce-Sandner et al., 1985). Consistent with our results, different authors have shown the activation of FFAR2 by BSCFAs and the differences in potency between isomers (Le Poul et al., 2003; Schmidt et al., 2011). A preliminary study has clearly demonstrated that 2MB and 3MB BSCFAs could induce bFFAR2-dependent β-arrestin 2 recruitment and MAP kinase ERK phosphorylation (Hudson et al., 2012). In the current study, we investigated 2MP, 2MB, and 3MB, which are C4:0 (2MP) and C5:0 (2MB and 3MB) isomers. 3MB behaved as a full agonist for both the recruitment and luciferase reporter assays, with potencies close to those of the reference ligand C6:0 and its own isomer C5:0. Interestingly, despite sharing the same molecular formula, changes in the methyl position, when considering 3MB and 2MB, significantly affected their capacity to activate bFFAR2.

We observed that 2MB behaved as a full agonist for Gα_i_ and Gα_q_ reporter gene assay but was a partial agonist for G protein (mGα_i_ and mGα_q_) and β-arrestin 2 recruitment. Furthermore, 2MB showed a significant decrease in potency, especially for β-arrestin 2 recruitment, compared to 3MB. The FFA C4:0 and its isomer 2MP also showed significant differences in the activation pattern of bFFAR2. C4:0 behaved more closely to the reference ligand, being classified as a full agonist for mGα_i_ and mGα_q_ recruitment and SRE gene transcription and having lower but similar potencies to C6:0. In contrast, 2MP behaved as a weak partial agonist for both G proteins and, in particular, β-arrestin 2 recruitment. These pharmacological differences between BSCFAs and their isomers might be the result of the molecular structure of these FFAs and their interaction with bFFAR2-binding sites. A recent study on another member of the FFAR family, FFAR4, demonstrated its capacity to assume diverse conformations as a function of the number and positioning of double bonds on the carbon skeleton of LCFAs (Mao et al., 2023), which might also occur in bFFAR2, although further studies are necessary to confirm this hypothesis.

Internalization of GPCRs from the PM and trafficking to intracellular compartments is essential for sorting GPCRs to recycling or degradative/lysosomal pathways. Recent studies have shown that GPCR signaling is not restricted to the PM but can also occur at the level of endosomes, as reviewed by Flores-Espinoza and Thomsen (2024). An additional level of complexity has been added by the discovery that GPCRs can be trafficked to different endocytic compartments. Internalized GPCRs are trafficked either to EEs, which are considered to be the primary sorting compartments (Hanyaloglu and Zastrow, 2008), or to the recently discovered very early endosome (VEE) (Jean-Alphonse et al., 2014). The process of GPCR trafficking to EE, VEE, or both varies from one GPCR to the next. A previous study has demonstrated that the human FFAR2 internalizes in both EE and VEE, with a marked preference for VEE upon C3:0 stimulation (Caengprasath et al., 2020).

The present study demonstrates that all FFAs studied induce bFFAR2 internalization from the PM. The observed signals exhibited a degree of alignment with our FFA classification in relation to full/partial agonism, whereby full agonists resulted in a greater degree of internalization than partial agonists. The majority of our ligands direct the internalized bFFAR2 toward EE, as evidenced by the BRET signal increases observed between bFFAR2 and the EE FYVE sensor. However, two ligands, 2MP and 3MB, exhibited contrasting behavior with regard to endocytic compartment localization, which was unexpected. 3MB induced a receptor internalization that corresponded to 100% of that observed for C6:0. However, when evaluating the trafficking to the EE, the 3MB response was only 30% of that of C6:0. The trafficking of bFFAR2 to EE was not observed when stimulated with 2MP, although marked bFFAR2 internalization from the PM was observed.

These findings might suggest that 2MP and 3MB facilitate the trafficking of bFFAR2 into distinct endocytic compartments. The present study indicates, for the first time, that endogenous agonists of a given GPCR may have the capacity to elicit disparate trafficking patterns. To date, there are no available BRET sensors for the detection of internalized GPCRs within VEE. Nevertheless, as it has been demonstrated that human FFAR2 does internalize within VEE (Caengprasath et al., 2020), we can hypothesize that 2MP and 3MB, to a lesser extent, reroute bFFAR2 trafficking from EE to VEE. This particular bFFAR2 trafficking pattern associated with 2MP and 3MB has the potential to influence cellular signaling. GPCR C-terminal tail modification to redirect GPCR trafficking to an unconventional endocytic compartment has been shown to result in alterations in the MAP kinase ERK phosphorylation temporal pattern (Jean-Alphonse et al., 2014). Furthermore, it is important to note that the bFFAR2 internalization and trafficking dynamics obtained in this study were measured using BRET sensors. Based on our analyses, it is unclear whether the low bFFAR2 trafficking to the EE, particularly when stimulated with 2MP, might be related to weak potencies and/or limitations of the BRET assay. Follow-up studies using other methods, such as confocal microscopy, might confirm our results.

A substantial body of research has indicated that BSCFAs possess distinctive physiological characteristics in dairy cows. Their relative concentration is indicative of rumen microbial diversity and protein diet composition (Wang et al., 2022). A recent study has identified 2MP as a potential indicator of heat stress in beef cattle (Correia Sales et al., 2021). A correlation has been established between BSCFAs’ concentration and various milk production parameters, including milk protein concentration and milk production (Papas et al., 1984; Peirce-Sandner et al., 1985; Wang et al., 2022). Furthermore, the supplementation of BSCFAs in dairy cows has been demonstrated to impact dairy cow physiology, including glucose dynamics and milk synthesis (Copelin et al., 2021; Gouveia et al., 2024). The present study suggests that BSCFAs exhibit specific pharmacological properties and trafficking patterns toward bFFAR2 compared to their isomers. It would, thus, be of interest to determine whether the response specificity of bFFAR2 following its activation by BSCFAs is central to the coordination of BSCFA-dependent physiological responses in dairy cows.

Overall, the present study reports different short- and medium-chain FFAs as bFFAR2 endogenous ligands. The activation of bFFAR2 by these ligands was demonstrated from upstream to downstream signaling pathways using HEK293A cells. We highlight the importance of species-specific studies for GPCRs as we observed distinct pharmacological properties of the bFFAR2 ligands compared with, for example, the human receptors. We also report BSCFAs’ specific properties on bFFAR2 trafficking to intracellular compartments. Our study represents a significant first step in understanding bFFAR2 and its potential relevance to bovine physiology. Future studies should investigate the biological outcomes of specific FFAs in activating bFFAR2 in different cell types and tissues, evaluating the potential use of this receptor as a pharmacological target in the context of health disorders in bovines.

## Data Availability

The original contributions presented in the study are included in the article/Supplementary Material; further inquiries can be directed to the corresponding author.
