# Supplementary material for: Endogenous ligands of bovine FFAR2/GPR43 display distinct pharmacological properties
Source: Front Cell Dev Biol. 2025 Aug 20;13:1645031. doi: 10.3389/fcell.2025.1645031 (PMC12405480; doi:10.3389/fcell.2025.1645031)
Supplement: Supplementary file 1 [file DataSheet1.docx]

Supplementary Material

# Suppl**ementary Figures**

**
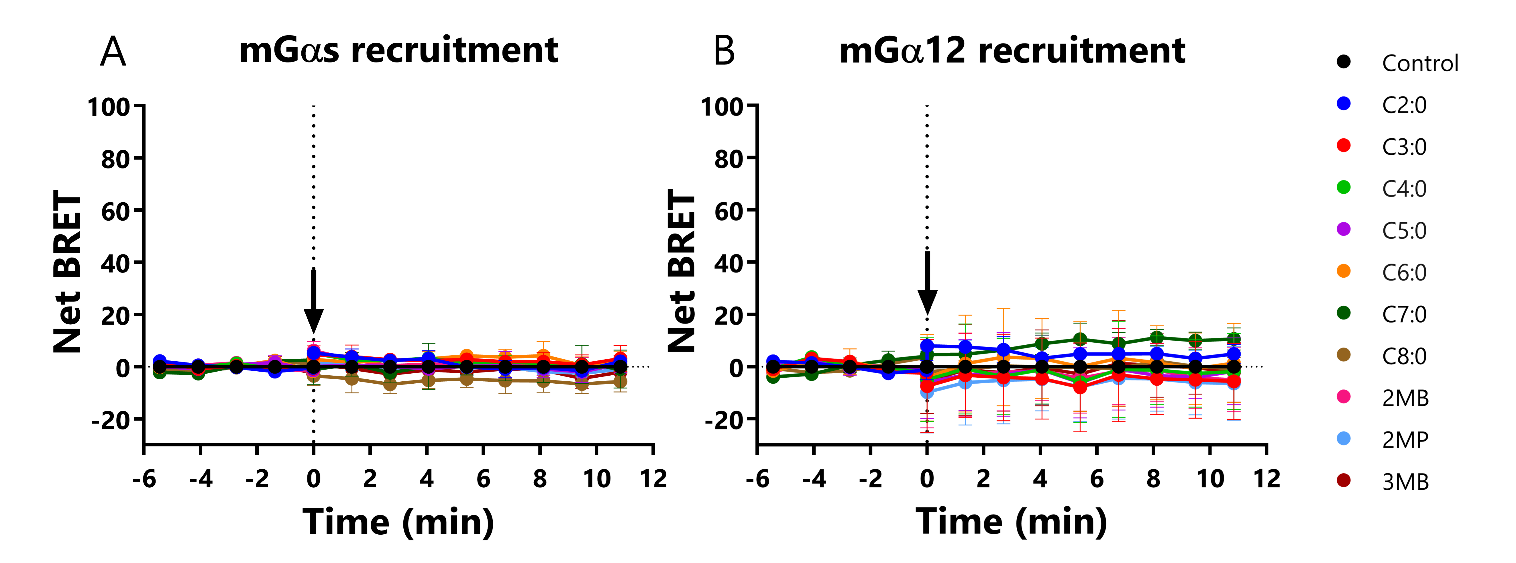
Supplementary Figure 1**. Kinetic curves of free fatty acids (FFAs)-stimulated HEK293A cells expressing bFFAR2. **(A)** mGα_s_, and **(B)** mGα_12_ recruitment to the receptor. Cells were stimulated (time = 0 min) with C2:0 at 0.1 M, C8:0 at 1 mM, and the other FFAs at 3.16 mM. BRET signals were monitored for 10 min after stimulation with FFAs and net BRET values were calculated by subtracting the ratio 530/30 over 480/30 nm emission of ligand stimulated cells from the same ratio of control cells, all multiplied by a constant 10^3^. Net BRET were plotted as a function of time. The results are shown as mean ± SD from at least three independent experiments.

**
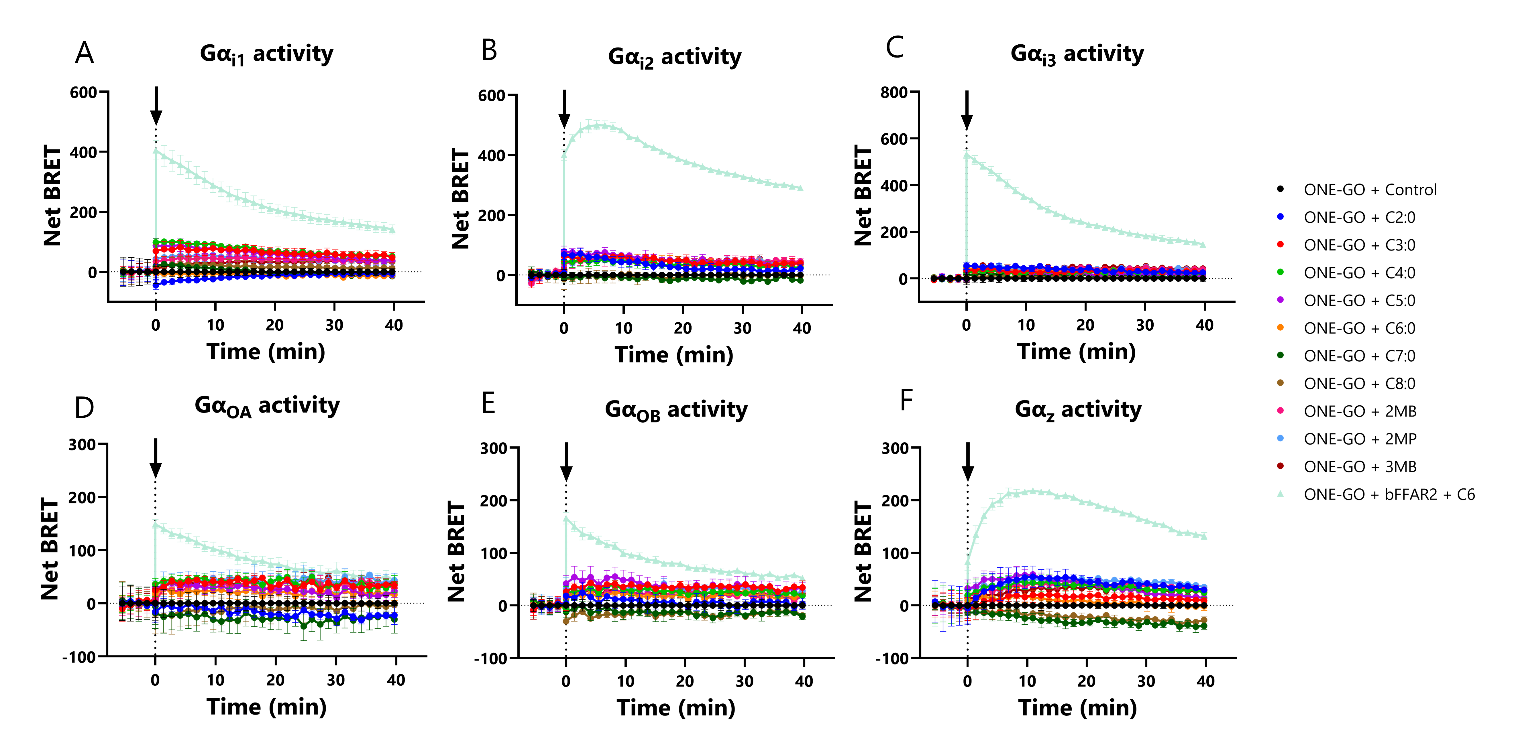
**

**Supplementary Figure 2**. Kinetic curves of free fatty acids (FFAs)-stimulated HEK293A cells. Measurement of **(A)** Gα_i1_, **(B)** Gα_i2_, **(C)** Gα_i3_, **(D)** Gα_OA_, **(E)** Gα_OB_, and **(F)** Gα_Z_ activity by Gα-GTP formation. Cells were transfected with ONE-GO biosensors alone (30 ng/well), or in addition to bFFAR2 (1:1 ratio, 30 ng/well; turquoise filled Δ). After 48 h, cells were stimulated (time = 0 min) with C2:0 at 0.1 M, C8:0 at 1 mM, and the other FFAs at 3.16 mM. BRET signals were monitored for 40 min after stimulation, and net BRET values were calculated by subtracting the ratio 535/15 over 460/40 nm emission of ligand stimulated cells from the same ratio of control cells, all multiplied by a constant 10^3^. Net BRET were plotted as a function of time. The results are shown as mean ± SD from at least three independent experiments.

**
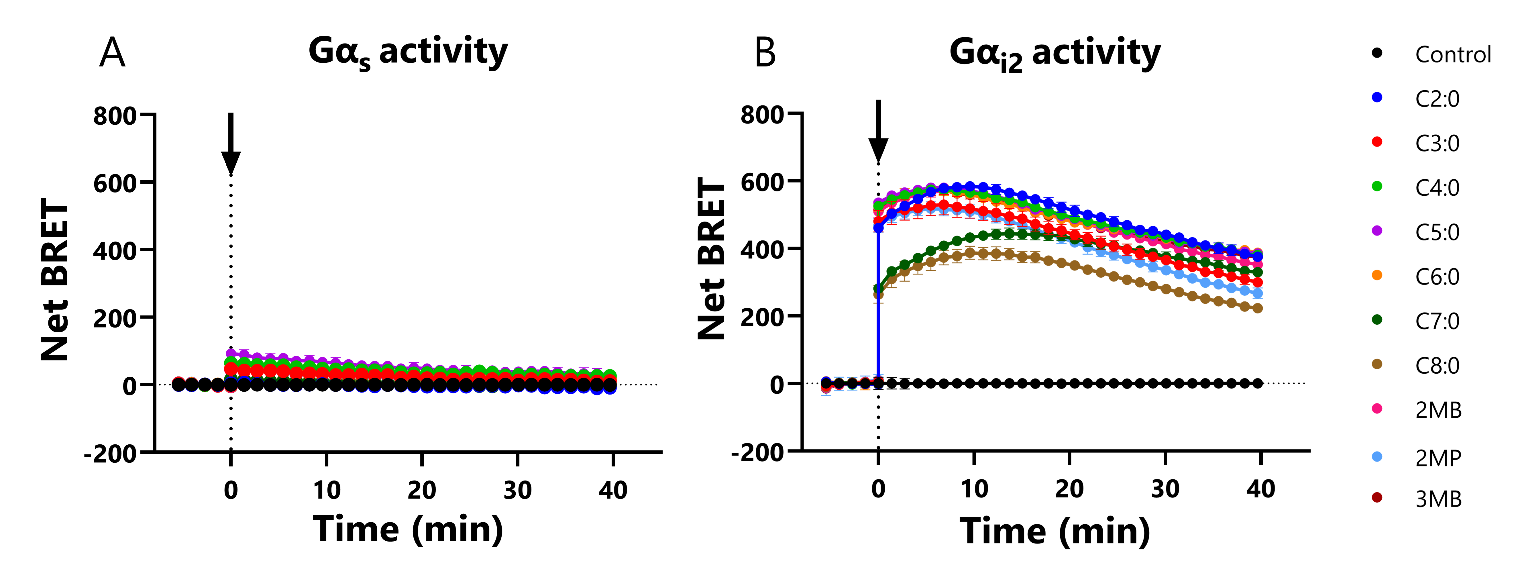
** **Supplementary Figure 3**. Kinetic curves of free fatty acids (FFAs)-stimulated HEK293A cells expressing bFFAR2 and **(A)** ONE-GO Gα_s_, and **(B)** ONE-GO mGα_i2_ biosensors. Cells were transfected with bFFAR2 and ONE-GO biosensors at a 1:1 ratio (30 ng/well). After 48 h, cells were stimulated (time = 0 min) with C2:0 at 0.1 M, C8:0 at 1 mM, and the other FFAs at 3.16 mM. BRET signals were monitored for 40 min after stimulation, and net BRET values were calculated by subtracting the ratio 535/15 over 460/40 nm emission of ligand stimulated cells from the same ratio of control cells, all multiplied by a constant 10^3^. Net BRET were plotted as a function of time. The results are shown as mean ± SD from at least three independent experiments.


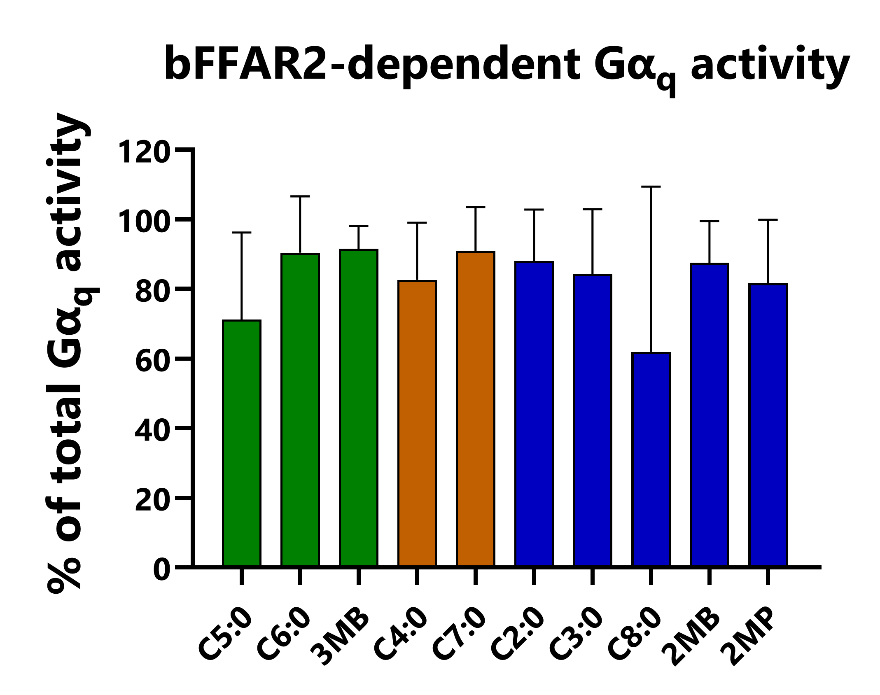


**Supplementary Figure 4**. bFFAR2-dependent Gα_q_ activity of free fatty acids (FFAs)-stimulated HEK293A cells. Cells were transfected with the ONE-GO Gα_q_ biosensor (30 ng/well), with or without the presence of bFFAR2 (30 ng/well). After 48 h, cells were stimulated with C2:0 at 0.1 M, C8:0 at 1 mM, and the other FFAs at 3.16 mM. BRET signals were monitored for 40 min after stimulation, and net BRET values were calculated by subtracting the ratio 535/15 over 460/40 nm emission of ligand stimulated cells from the same ratio of control cells, all multiplied by a constant 10^3^. Area under the curves (AUC) were generated for each ligand. bFFAR2-dependent Gα_q_ activity was calculated by determining the AUC for cells transfected with both ONE-GO Gα_q_ biosensor and bFFAR2 receptor (total Gα_q_ activity, 100%), substracted by the AUC for cells transfected with the ONE-GO Gα_q_ biosensor alone (bFFAR2-independent Gα_q_ activity). The results are shown as percentage ± SD from at least three independent experiments. Color coding represents the classification free fatty acids into 3 different groups based on their maximum response towards three different recruitment assays (mGα_q_, mGα_i_, and β-arrestin 2): A. full agonists (green; C5:0, C6:0 and 3MB), B. partial/full agonists (orange; C4:0 and C7:0), and C. partial agonists (blue; C2:0, C3:0, C8:0, 2MB, and 2MP).


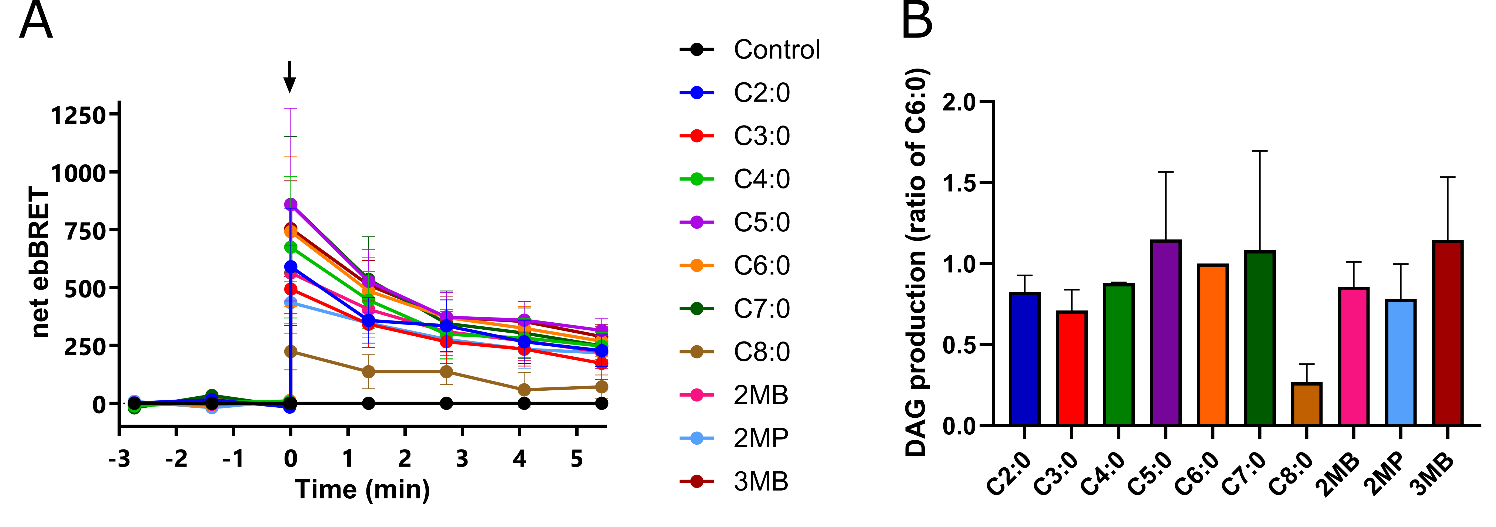
**Supplementary Figure 5**. Diacylglycerol (DAG) production of free fatty acids (FFAs)-stimulated HEK293A cells expressing bFFAR2. Cells were transfected with DAG sensor (Rluc_C1B) (3 ng/well), rGFP-CAAX biosensors (30 ng/well) and bFFAR2 (30 ng/well). After 48 h, cells were stimulated with C2:0 at 0.1 M, C8:0 at 1 mM, and the other FFAs at 3.16 mM. Enhanced bystander BRET (ebBRET) signals were monitored for 5 min 30 s after stimulation, and net ebBRET values were calculated by subtracting the ratio 535/15 over 410/80 nm emission of ligand stimulated cells from the same ratio of control cells, all multiplied by a constant 10^3^. **(A)** Net ebBRET were plotted as a function of time. The results are shown as mean ± SD from at least three independent experiments. **(B)** Area under the curve (AUC) were calculated from net ebBRET results and normalized as ratio of C6:0. The results are shown as mean ± SD from at least three independent experiments.
